# Exploring the Frontiers of Neuroinflammation: New Horizons in Research and Treatment

**DOI:** 10.3390/cimb46100692

**Published:** 2024-10-19

**Authors:** Giovanna Rigillo, Silvia Alboni

**Affiliations:** 1 Department of Biomedical, Metabolic and Neural Sciences, University of Modena and Reggio Emilia, 41125 Modena, Italy; 2 Department of Life Science, University of Modena and Reggio Emilia, 41125 Modena, Italy

The Special Issue “*Advanced Research in Neuroinflammation*” offers a rich and diverse collection of studies that deepen our understanding of how inflammatory mediators are involved in various neurological conditions. The Special Issue includes original studies and review articles exploring the complex interplay between immune responses and neuronal health and proposes novel therapeutic strategies to modulate neuroinflammation, aiming to improve outcomes for patients suffering from spinal cord injuries to neurodegenerative disorders.

An increasing body of evidence confirms the crucial role of inflammation mediators in determining the state of health or disease in central disorders. Inflammatory signaling pathways regulate physiological processes such as cell survival, differentiation, and metabolism.

As highlighted by the studies collected in this Special Issue, both the activation and inhibition of the inflammatory response can contribute to developing central nervous system (CNS) disorders [1], including by altering the ability of the organism to respond to damage.

The contribution of Di Santo et al. on ischemic brain tolerance provides an exciting exploration into how preconditioning the brain can modulate neuroinflammatory responses and protect against ischemic damage [2]. In this study, the authors also highlight a key factor, and that is that the neuro–immune–endocrine response triggered by environmental stimuli depends on the health status and individual risk factors. The different responses to inflammatory stimuli likely depend on the activation level of inflammatory signaling pathways.

In this context, Vavougios et al. investigate the long-term effects of SARS-CoV-2 on olfactory networks and its implications for Alzheimer’s disease, with a focus on type I interferon signaling. They found a 14-gene signature associated with SARS-CoV-2 infection and a response to Alzheimer’s disease pathology [3].

Virtually all cell types that make up our brain (i.e., neurons, microglia, astrocytes, and oligodendrocytes) participate in determining the ‘levels of inflammatory signaling activation,’ thus modulating central functions and the neuronal framework, and contributing to determining the health status of the CNS.

In this context, increasing attention is being focused on gaining astrocytes [4,5]. Ho et al., by expressing the mutant human G2019S of the Leucine-rich repeat kinase 2 (LRRK2) gene in astrocytes, show that this mutation reduces cell viability, increases the expression of proinflammatory cytokines, and promotes astrogliosis. Moreover, treatment with the conditioned astrocyte medium expressing the mutated gene disrupts the dopamine synthesis pathway and decreases the cell viability of rat dopaminergic neurons, thus suggesting a role for this mutation in Parkinson’s disease progression [6].

Unrevealing the role of inflammation mediators in the etiopathogenesis and progression of CNS diseases is crucial for developing new, more effective, and safer therapeutic approaches. For instance, the review of Terracina et al. investigates how nerve growth factors (NGFs) interact with autoimmune pathways to exacerbate inflammation in autoimmune diseases, highlighting the potential for targeted interventions that could disrupt this harmful cycle [7]. This research sheds light on new molecular targets that could be crucial for controlling inflammation in diseases where immune dysregulation is central to pathology.

Interestingly, in the study of Li et al. nicotinamide riboside (NR), a nicotinamide adenine dinucleotide (NAD^+^) precursor, supplementation is suggested as a potential treatment following spinal cord injury (SCI).

Later research demonstrates that NR supplementation modulates chemotaxis and reduces inflammation after SCI. By decreasing inflammation, NR not only ameliorates immediate damage but also facilitates functional recovery, offering a promising avenue for treating spinal cord injuries, where inflammation is a major barrier to healing.

In the context of Alzheimer’s disease, Hickey et al. explore how cannabidiol (CBD) modulates oxidative stress and neuroinflammation [8,9]. Their research provides evidence that CBD, by targeting specific inflammatory pathways, holds potential as a therapeutic agent in slowing the progression of Alzheimer’s disease and other neurodegenerative disorders, where inflammation plays a crucial role in neuronal death.

The contribution of the endocannabinoid system and cannabinoids in neuroprotection was also reviewed by Carter et al. about stroke pathogenesis. The authors present a dual perspective: while specific cannabinoids like CBD may offer neuroprotective benefits, THC-containing cannabis use, especially in younger populations, is increasingly associated with a higher risk of ischemic stroke. The review underlines that cannabinoid receptors, particularly CB1 and CB2, play a pivotal role in neuroprotection by reducing neuronal damage and excitotoxicity. However, the same receptors are also implicated in vascular dysfunction, contributing to the risk of stroke [10].

Finally, the systematic review by Panaitescu et al. delves into the impact of gut microbiota on neuroinflammation and motor function in Parkinson’s disease [11]. Their work describes the growing evidence that gut–brain interactions are crucial for understanding the inflammatory pathways that drive neurodegeneration and points to the microbiome as a potential therapeutic target.

Overall, the articles of this Special Issue underscore the central role of neuroinflammation in a wide range of neurological disorders, spanning spinal cord injury, CNS infection, and neurodegenerative diseases, and highlight the complexity of neuroinflammation as both a pathological driver and a potential therapeutic target.

This editorial provides an overview of the central findings from the Special Issue, capturing the essence of how neuroinflammation research is evolving to offer new therapeutic possibilities for a range of neurological disorders.
